# Global Characterization of Fungal Mitogenomes: New Insights on Genomic Diversity and Dynamism of Coding Genes and Accessory Elements

**DOI:** 10.3389/fmicb.2021.787283

**Published:** 2021-12-01

**Authors:** Paula L. C. Fonseca, Ruth B. De-Paula, Daniel S. Araújo, Luiz Marcelo Ribeiro Tomé, Thairine Mendes-Pereira, Wenderson Felipe Costa Rodrigues, Luiz-Eduardo Del-Bem, Eric R. G. R. Aguiar, Aristóteles Góes-Neto

**Affiliations:** ^1^Department of Genetics, Ecology and Evolution, Universidade Federal de Minas Gerais, Belo Horizonte, Brazil; ^2^Department of Biological Science (DCB), Center of Biotechnology and Genetics (CBG), Universidade Estadual de Santa Cruz (UESC), Ilhéus, Brazil; ^3^Graduate School of Biomedical Sciences, Baylor College of Medicine, Houston, TX, United States; ^4^Program in Bioinformatics, Loyola University Chicago, Chicago, IL, United States; ^5^Molecular and Computational Biology of Fungi Laboratory, Department of Microbiology, Instituto de Ciências Biológicas, Universidade Federal de Minas Gerais, Belo Horizonte, Brazil; ^6^Program of Bioinformatics, Instituto de Ciências Biológicas, Universidade Federal de Minas Gerais, Belo Horizonte, Brazil; ^7^Department of Botany, Instituto de Ciências Biológicas, Universidade Federal de Minas Gerais, Belo Horizonte, Brazil

**Keywords:** mitochondrial genome, homing endonuclease, intron, size variation, comparative genomics

## Abstract

Fungi comprise a great diversity of species with distinct ecological functions and lifestyles. Similar to other eukaryotes, fungi rely on interactions with prokaryotes and one of the most important symbiotic events was the acquisition of mitochondria. Mitochondria are organelles found in eukaryotic cells whose main function is to generate energy through aerobic respiration. Mitogenomes (mtDNAs) are double-stranded circular or linear DNA from mitochondria that may contain core genes and accessory elements that can be replicated, transcribed, and independently translated from the nuclear genome. Despite their importance, investigative studies on the diversity of fungal mitogenomes are scarce. Herein, we have evaluated 788 curated fungal mitogenomes available at NCBI database to assess discrepancies and similarities among them and to better understand the mechanisms involved in fungal mtDNAs variability. From a total of 12 fungal phyla, four do not have any representative with available mitogenomes, which highlights the underrepresentation of some groups in the current available data. We selected representative and non-redundant mitogenomes based on the threshold of 90% similarity, eliminating 81 mtDNAs. Comparative analyses revealed considerable size variability of mtDNAs with a difference of up to 260 kb in length. Furthermore, variation in mitogenome length and genomic composition are generally related to the number and length of accessory elements (introns, HEGs, and uORFs). We identified an overall average of 8.0 (0–39) introns, 8.0 (0–100) HEGs, and 8.2 (0–102) uORFs per genome, with high variation among phyla. Even though the length of the core protein-coding genes is considerably conserved, approximately 36.3% of the mitogenomes evaluated have at least one of the 14 core coding genes absent. Also, our results revealed that there is not even a single gene shared among all mitogenomes. Other unusual genes in mitogenomes were also detected in many mitogenomes, such as *dpo* and *rpo*, and displayed diverse evolutionary histories. Altogether, the results presented in this study suggest that fungal mitogenomes are diverse, contain accessory elements and are absent of a conserved gene that can be used for the taxonomic classification of the Kingdom Fungi.

## Introduction

The Kingdom Fungi is one of the most diverse and globally distributed groups of eukaryotes. Fungi evolved a wide morphological, physiological, and ecological heterogeneity that allowed them to perform vital functions in both terrestrial and aquatic ecosystems (Walker and White, 2005; James et al., 2020; Li et al., 2021). The global fungal diversity is estimated between 2 to 5 million species, that are further classified into 12 phyla: Ascomycota, Basidiomycota, Entorrhizomycota, Chytridiomycota, Monoble-pharidomycota, Neocallimastigomycota, Mucoromycota, Zoop agomycota, Blastocladiomycota, Aphelidiomycota, Crypto mycota/Rozellomycota, and Microsporidia (James et al., 2020; Li et al., 2021).

Estimations indicate that only ∼10% of the fungal diversity has been described, with the subkingdom Dikarya containing ∼97% of all known species, further distributed into the phyla Ascomycota, Basidiomycota, and the recently added Entorrhizomycota (James et al., 2020; Li et al., 2021). Ascomycota alone encompasses ∼70% of all Dikarya diversity (Schmidt-Dannert, 2016), suggesting that most groups of fungi are still underrepresented. Thus, integrative studies to identify and describe new species, as well as those applying large phylogenies and sequencing of genomes (both nuclear and mitochondrial), are still needed to better understand the evolution, biology, and phylogeny of this highly important group of organisms.

Eukaryotes and prokaryotes are in constant interaction, and the most ancient symbiosis we know about led to the emergence of mitochondria (Carvalho et al., 2015). Mitochondria are bi-membranous cytoplasmic organelles responsible for a vast range of functions. Their central function is the production of energy, via oxidative phosphorylation and adenosine triphosphate (ATP) synthesis (Kulik et al., 2021). Mitochondria also produce metabolic precursors of macromolecules, such as proteins and lipids, and metabolic by-products, such as ammonia and reactive oxygen species (Spinelli and Haigis, 2018). In addition, these organelles are involved in apoptosis, homeostasis maintenance, and stress response (Grahl et al., 2012; Verma et al., 2018; Zardoya, 2020). It is proposed that mitochondria originated from the endosymbiosis of an ancestral free-living Alphaproteobacteria, which was permanently integrated into the host cell (Carvalho et al., 2015; Muñoz-Gómez et al., 2017). Mitochondria contain their own genome (mitochondrial DNA – mtDNA or mitogenome), with independent replication and inheritance. Throughout evolutionary history, most of the mitochondrial early genes have been transferred to the nuclear genome. As a result, most mitochondrial proteins are encoded by the nucleus, translated by ribosomes in the cytosol, and subsequently transferred into the mitochondria. Therefore, extant mtDNA contains mainly protein-coding genes that play central roles in oxidative phosphorylation (Verma et al., 2018). The mitogenome and the nuclear genome are in constant communication, during which the mtDNA is responsible for the stability of the nuclear genome and proper cell functioning. The mitogenome also depends on the nuclear genome to maintain its functionality, integrity, and stability (Kaniak-Golik and Skoneczna, 2015; Kulik et al., 2021).

The architecture of mitogenomes can vary among eukaryotes. In fungi, mitogenomes can be linear or circular. Circular mitogenomes are by far the most common, while linear mitogenomes are mainly described in yeasts, such as *Saccharomyces cerevisiae* (Ascomycota) (Williamson, 2002; Formaggioni et al., 2021). Typical fungal mitogenomes such as those belonging to Ascomycota and Basidiomycota (Dikarya Subkingdom) usually display a core genome with 14 conserved protein-coding genes (*atp6*, *8*, and *9; cob*; *cox1– 3;* and *nad1– 6, 4L*), the ribosomal genes *rnl* and *rns*, and from 20 and 31 tRNA genes (Zardoya, 2020; Kulik et al., 2021). The genomes belonging to subkingdom Dikarya also have the ribosomal protein gene *rps3* that is lacking in some taxa, such as the genus *Candida* (Freel et al., 2015; Formaggioni et al., 2021). Mitogenome length is also quite variable among fungal taxa, ranging from 12 to 272 kbp in *Rozella allomycis* and *Morchella crassipes*, respectively (James et al., 2013; Liu et al., 2020). This plasticity in mtDNA is related to factors like the length and number of introns, intergenic regions, repetitive DNA, plasmid insertions, segment duplications, Open Reading Frames (ORFs) without defined function (uORF), and homing endonuclease genes (HEG) (Fonseca et al., 2020; Zardoya, 2020; Araújo et al., 2021; Kulik et al., 2021).

The presence of accessory DNA elements, such as introns, HEGs, and uORFs are directly correlated to the modulation of mtDNA (Fonseca et al., 2020; Araújo et al., 2021). In fungal mitogenomes, group I and group II introns can be identified. The classification of these introns is based on sequence conservation, secondary structure configuration, and helix shape (Hausner et al., 2014). Group I introns are the most frequent and can encode HEGs families like LAGLIDADG and GIY-YIG, which are related to transposition of sequences in different regions of the mitogenome (Hafez and Hausner, 2012; Kulik et al., 2021). Group II introns differ from type I in structure, sequence, and splicing mechanism. The distribution of introns among different species is considered irregular, and the same introns and their associated HEGs can be identified in mitogenomes that are considered evolutionarily distant (Hausner, 2003; Lang et al., 2007; Hausner et al., 2014; Zardoya, 2020).

In spite of the diversity of size and genes, mitogenomes can be a key tool to investigate phylogenetic relationships by exhibiting a low recombination rate, diverse patterns of inheritance, and by displaying a mutation rate different from the nuclear genome (Rubinoff and Holland, 2005; Mendoza et al., 2020). Additionally, the high number of copies inside the cytoplasm of the cell facilitates the amplification and sequencing of partial or complete mitogenomes (Kulik et al., 2021). Nonetheless, the sequencing of mitogenomes from a larger number of fungal species is necessary to assess the presence of a universal marker to understand the evolution of major fungal lineages (Kulik et al., 2015; Smith, 2015; Avin et al., 2017).

Considering the increasing number of fungal mitogenomes available in genomic databases in the last few years due to exponential growth of high-throughput sequencing (HTS), we observed a discrepancy regarding the diversity of fungal taxa represented. As fungi display a great diversity of species with a broad range of ecological functions, we selected the Kingdom Fungi as a model to better understand the macroevolutionary patterns underlying mitogenome diversity. We characterized the fungal mitogenomes according to genome size, content, and sequence homology to better understand the mechanisms involved in the observed sequence diversity among fungal mtDNAs. We also correlated the genetic code with mitogenome topology, genome size, and gene composition of core and accessory elements. Some species of the 12 fungal phyla are overrepresented; meanwhile, at least four phyla have no representative in genomic databases. In addition, we found a high variation in mitogenome length in fungi. This variation may be due to the variable abundance of accessory (introns, HEGs and uORFs) and protein-coding regions. Our analyses showed that no gene is universally conserved in fungal mitogenomes, suggesting that mitochondrial gene content can vary widely without disrupting organelle function. The data explored herein provides a significant addition to our understanding of the diversity and evolution of fungal mitogenomes.

## Materials and Methods

### Bibliographic Survey for Fungal Mitogenomes Studies

A literature review was carried out to obtain information on the number and objective of studies published with fungal mitogenomes until December 2020. The search was performed with the keywords “((fungi[Title/Abstract]) OR (fungal[Title/Abstract])) AND ((mitochondria[Title/Abstract]) OR (mitochondria[Title/Abstract])) AND genomic” in the PubMed database. The abstract of each study was evaluated and classified by year of publication and by topic: characterization of mitogenomes (description of a new mitogenome, comparative analyses) or biotechnological (study of mitogenomes in industrial or antifungal processes). The Supplementary Table 1 presents all data from the resulting articles.

### Inclusion Criteria of Mitogenomes

A total of 788 mitogenomes were available for download in the NCBI database^1^ on December 4th, 2020. In our analyses, we selected representative and non-redundant mitogenomes based on the threshold of 90% identity using the CD-HIT software (Li and Godzik, 2006). This threshold was defined after a comparison of the results obtained with 70, 80, and 90–99% similarity among the genomes (Supplementary Table 2). Two mitogenomes (NC_003061.1 and NC_003060.1), classified in NCBI as complete but with less than 2 kbp in size, were removed. Another 22 mitogenomes of poor quality containing many gaps (more than 20% of N bases) were also removed from our analyses. The selected mitogenomes (*n* = 685) were used in all subsequent analyses. Identification of fungal species, taxonomic classification, and accession numbers for the mitogenomes are provided in Supplementary Table 3.

### Mitochondrial Genome Annotation

We verified each species’ page in NCBI Taxonomy Browser^2^ to assess the proper mitochondrial genetic code for protein-coding gene annotation. Mitogenomes were annotated using Mfannot^3^ to standardize the process and to allow a fairer comparative analysis. Mfannot program was selected for its greater specificity in the annotation of fungal mitochondrial genes that has already been used in other studies of mitogenome characterization (Chen et al., 2019; Fonseca et al., 2020; Araújo et al., 2021). Mitogenomes that did not display genes considered essential (*rns*, *rnl*, *rps3*, *atp*, *cox* and *nad* families, and *cob*) were manually curated. A gene was considered absent when it did not show nucleotide similarity above 50% when compared to all mitochondrial genes deposited in the NCBI database^4^. All annotations are available in Supplementary File 1.

### Annotation of uORFs

With the annotation files generated by Mfannot, we extracted the sequences annotated as “ORFs” and translated them into amino acids using the corresponding mitochondrial genetic code for each species. These protein sequences were then submitted to the Batch CD-Search interface of NCBI’s Conserved Domain Database^5^ to identify known protein domains in the unidentified open reading frames.

Sequences that did not exhibit similarity with known domains were considered as uORFs and clustered into a single file. Sequences that presented identity above 80% with HEGs, DNA-polymerase (*dpo*), and RNA-polymerase (*rpo*), were clustered into different files for further analyses. Furthermore, we estimated the coding potential of all annotated ORFs (with and without known protein domains) using the CPC2 software (Kang et al., 2017). The ORF sequences classified as *rpo* or *dpo* were aligned with MAFFT (Katoh et al., 2019). The selection of the best nucleotide substitution model was performed using MEGA X software (Kumar et al., 2018) based on AIC criteria (Akaike, 1973). The best model selected for both datasets was GTRCAT. A maximum likelihood phylogeny with 1,000 bootstrap replicates was generated using MEGA X. The output files were used to plot the trees using the *ggtree* package v.3.0.2 (Yu, 2020) in R (R Core Team, 2021).

### Comparative Mitogenome Analysis

The comparative analysis consisted of evaluating the difference in length of mitogenomes of each phylum, the number of genes, the presence of core genes, the presence and length of introns, HEGs, uORFs, GC content, and similarity amongst the mitogenomes. We have developed in-house scripts to parse software outputs and calculate genome summary statistics to facilitate tabulating the data.

Initially, we wrote a Python script that uses Biopython’s modules to compute the length and GC content of every mitogenome analyzed in this study. Using the output files from Mfannot annotation, we designed a Bash script to generate tabular files containing the annotation data (“feature name,” “start coordinate,” “end coordinate,” “feature size,” and “feature strand”). According to “feature name,” output files (tabular and fasta format) were generated for multiple categories: ORFs, introns, HEGs, core genes (including the *rps3* ribosomal protein), tRNA genes and other unusual genes found in mitogenomes (*dpo* and *rpo*). The number of features per file was counted to get the total number of features per category. Another Python algorithm that removes overlaps of feature coordinates and calculates final genic lengths was written to properly calculate the length of the intergenic spaces in fungal mitogenomes. These numbers were subtracted from the total fungal mitochondrial genome length, resulting in the total length of intergenic regions. The data obtained from the aforementioned scripts were organized and plotted using the *ggplot2* package v.3.3.5 (Wickham, 2016) in R (R Core Team, 2021).

### Mitogenome Similarity Network Analyses

For the similarity network analyses, we first used BLASTn (Johnson et al., 2008) to assess similarity among mitogenomes. Then, we built two different networks: the first one consisted of only overrepresented mitogenomes (species that present more than three mitogenomes after the similarity filter made by CD-HIT), and the second one with all non-redundant mitogenomes used in this study, including the mitogenomes from the first network. In cases in which mitogenome pairs aligned in more than one segment, that is, the same pair aligned multiple times in different regions, we only considered the similarity index of the longest continuous alignment. Subsequently, we built the networks using the *ggraph* package v.2.0.5 (Pedersen and RStudio, 2021) in R (R Core Team, 2021).

## Results and Discussion

### Fungal Mitogenomes Overview

Fungal mitogenomes can be very informative to describe new species, identify genes related to fungicides, and to help understand the process of energy production and general cell physiology (Kulik et al., 2021). Although the genetic study of fungal mitochondria is of great relevance, literature about this topic is far from abundant (481 papers found in the last 40 years in our bibliographic search) (Supplementary Table 1). Most published articles are related only to the description of a new mitogenome or comparative mitogenomes analysis from a particular group of fungi, while a few others propose mitochondrial genes as molecular markers or the importance of mitogenomes for specific fungal species. Comparatively, according to Smith (2015), until 2015 more than 1,000 articles had been published for other kingdoms addressing issues of mitogenome description, use of mitochondrial information as a molecular marker, and origin and ancestry of these organisms (Smith, 2015). Considering the PubMed database, using the keyword Mitogenome or Mitochondrial genome, 5,302 articles are listed, of which 3,267 were published from 2016 to date.

**FIGURE 1:**
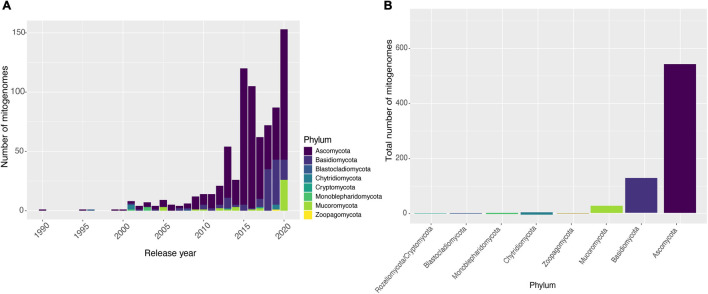
Availability of fungal mitogenomes in NCBI databases. **(A)** Number of mitogenomes published by year and classified by phyla, from 1990 to 2020. **(B)** Number of mitogenomes available for each phylum of the Kingdom Fungi.

Until December 4th, 2020, there were 788 fungal mitogenomes available in the NCBI database. The vast majority were deposited in the last 8 years (Figure 1A). The increase of mitogenomes in the last years can be explained by the emergence and improvements of HTS. However, the vast majority of the 147,933 identified fungal species (Kirk, 2020) still do not have their mitogenomes sequenced (Cheek et al., 2020). The number of mitogenomes available in the database does not reflect the fungal diversity, suggesting that further sequencing studies should be carried out to investigate the genomic architecture of the kingdom. Compared to Metazoa by the year 2014, there were already more than 4,000 complete mitogenome entries available in NCBI, representing more than 90% of all mitogenomes available in the database.

Different biases in fungal genomic analyses have been reported, with the most important regarding the substantially higher number of studies on Ascomycota species compared to species from other phyla. It is estimated that at least 63% of fungal species belong to Ascomycota (Kirk, 2020) although an even higher proportion of the fungal genomics literature is dedicated to this phylum. This may be due to the fact that this phylum comprises the largest number of species with high economic importance and diverse ecological functions, and species considered as model organisms, providing new information regarding the biology and evolution of the Kingdom Fungi (Robbertse et al., 2006; Schoch et al., 2009; Wallen and Perlin, 2018). Our search reflected this bias for fungal mitochondrial DNA sequences: from the total of 788 NCBI entries, Ascomycota has twice as many deposits as other phyla, with many species displaying more than one sequenced mitogenome. Moreover, some phyla are poorly sampled, such as Blastocladiomycota (two mitogenomes) and Zoopagomycota (three mitogenomes) (Figure 1B). This low sampling of the other phyla limits our knowledge on the composition and evolution of fungal mitogenomes. Nevertheless, it should still be considered that some fungal phyla have a much smaller number of species described when compared to Ascomycota and Basidiomycota, as is the case of Cryptomycota/Rozellomycota (Lazarus and James, 2015; Letcher and Powell, 2018; Li et al., 2021).

In this study, we aimed to evaluate the differences and similarities among the mitogenomes of the Kingdom Fungi using the CD-HIT software to select a dataset without the presence of highly similar sequences. At a 90% similarity, the first inflection point was observed, in which there was a reduction of the dataset by 10.28% (removal of 81 access numbers). Despite the removal of these sequences, the dataset remained with almost 90% of all mitogenomes, suggesting that the genomic content is quite divergent, which led us to investigate the conservation of protein-coding genes and the presence of accessory elements (introns, HEGs and uORFs) in most of the mitogenomes.

### Plasticity of Fungal Mitogenomes

#### Mitogenome Length Variation and Gene Conservation

**FIGURE 2:**
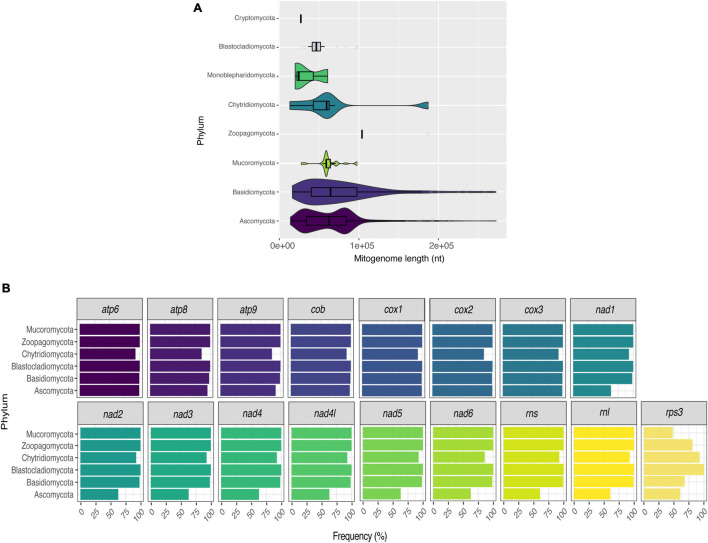
Size and composition of fungal mitogenomes. Size **(A)** and frequency of core genes **(B)** of fungal mitogenomes by phyla.

Many studies have shown that fungal mitogenomes have great structural plasticity (Fonseca et al., 2020; Araújo et al., 2021; Kulik et al., 2021). In this study, the length difference among mitogenomes ranges from 12 (*R. allomycis* Chytridiomycota – NC_003061) to 272 kbp (*Ophiocordyceps camponoti* Ascomycota – CM022976). Since the two mitogenomes are from different and distant phyla, we assessed whether mitogenome length would be related to phyla classification (Figure 2A). In general, Ascomycota (63,888 ± 35,298 bp) and Basidiomycota (77,394 ± 49,928 bp) mitogenomes have quite variable lengths. However, Cryptomycota (25,401 bp) and Mucoromycota (61,936 ± 14,895 bp) have smaller and more homogeneous sizes. This result may be related to the availability of data, since more than 90% are composed of mitogenomes from Ascomycota and Basidiomycota, while Mucoromycota and Cryptomycota represent 3.38% and 0.13% of mitogenomes, respectively.

**FIGURE 3:**
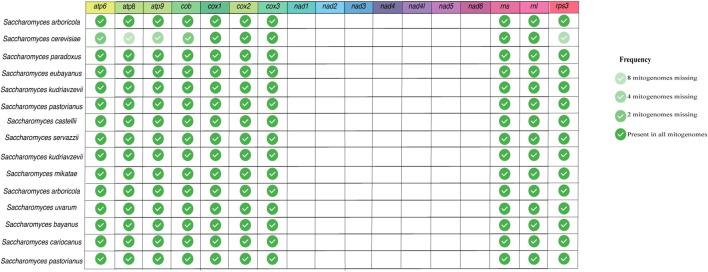
Presence of core genes in *Saccharomyces* mitogenomes. Fourteen protein-coding and two ribosomal genes were evaluated in the 160 curated *Saccharomyces* mitogenomes.

As the length of mitogenomes is variable and is related to variations in both protein-coding and accessory element regions (Paquin et al., 1997; Bullerwell, 2003; Al-Reedy et al., 2012; Araújo et al., 2021), we re-annotated all mitogenomes to facilitate the comparison between the features present in each genome. After the annotation, we estimated the presence/absence of mitochondrial genes that are considered essential in fungal mitogenomes. Several previous studies have already demonstrated the presence of *atp*, *cox*, *cob*, *nad*, *rns*, and *rnl* in fungal mitogenomes (Song et al., 2020; Kulik et al., 2021). According to our analyses, although most of the mitogenomes have all the previously expected core genes, there was not a single gene shared in all evaluated fungal species. Figure 2B displays the frequency of each gene per phylum. In this figure, the phylum Cryptomycota is not shown, as it has only one representative (*Paramicrosporidium saccamoebae* – CM008827) containing all genes except for *nad6*. Genes of *atp*, *cox*, and *cob* families are the most frequent among fungal mitogenomes. Homologs of *nad*, *rns* and *rnl* are the least found within Ascomycota. It is worth noting that 149 mitogenomes are from the genus *Saccharomyces*, which does not have *nad* homologs (Ruan et al., 2017). In addition, some *S. cerevisiae* mitogenomes also have no homologs of *atp* and *cob* (Figure 3). Many studies have already reported that the coding content of the genus *Saccharomyces* is well conserved (Freel et al., 2015; Ruan et al., 2017). No member of *Saccharomycetaceae* were shown to have *nad* homologs, and studies suggested that *nad* homologs were lost after the divergence of this family (Dujon, 2010).

As a controversy of the relationship between size and presence of core genes, the mitogenome of the yeast *Malassezia furfur* (CP046241) is 43 kb long but does not show any of the core genes. This mitogenome has 47 ORFs, of which 22 have unknown protein domains, and the others have domains from the superfamilies haloacid dehydrogenase (HAD), Glutenin high molecular weight, among others (Supplementary File 2). The species *M. furfur* is a basidiomycotan yeast of medical importance as it can cause skin and blood infections (Theelen et al., 2018), and the mitogenome used in this study was previously used for taxonomic classification of the species (Sankaranarayanan et al., 2020). The genes found in this mitogenome, despite having mitochondrial-related functions, are not commonly found in mitogenomes, but in nuclear genomes (Theelen et al., 2018). Another species lacking many mitochondrial genes is *Rozella allomyces* which has the smallest mitogenome size in our dataset (12 kbp), and lacks nine core genes (*atp6*, *nad1-6*, and *rnl*).

During evolution, many mitochondrial genes migrated to the nuclear genome. In fungi, the majority of genes related to mitochondrial function are found in the nuclear genome (Bolender et al., 2008). The main theory for the mechanism involved in the escape of DNA to the cytosol and, consequently, to the nucleus states that these genetic fragments are transferred by mobile elements that use the non-homologous (NHEJ) machinery for integration (Berg and Kurland, 2000; Tsuji et al., 2012). In *S. cerevisiae*, transfer of mtDNA to the nuclear genome has already been observed when the cell had mutations in specific nuclear genes, depending on the structure of the mitogenome and availability of sugars to allow fermentation in the medium, which could lead to the high variation of phenotypes to anaerobic environments (Shafer et al., 1999; Peter et al., 2018). The absence of core genes in the species evaluated may be an indication that these genes migrated to the nuclear genome. Studies exploring the nuclear genome of these species must be carried out to confirm the presence of genes of mitochondrial origin in the nuclear genome.

#### Presence and Abundance of Non-coding Elements

After assessing the number and length of the following accessory elements: introns, HEGs, and uORFs, in all the fungal mitogenomes filtered by CD-HIT, we characterized the number of HEGs found per genome and grouped by phylum. Many studies have been discussing the presence and influence of non-coding elements in mitogenomes of different fungal groups (Fonseca et al., 2020; Araújo et al., 2021; Kulik et al., 2021). HEGs are classified as selfish elements that can transpose by breaking the DNA at specific sites, which consequently generates gene conversion events. These accessory elements can be found in introns or as free-standing, which ensures their dissemination and fixation in the mitogenome and may even alter the reading frame of protein-coding genes (Hausner, 2003; Barzel et al., 2011; Stoddard, 2014).

**FIGURE 4:**
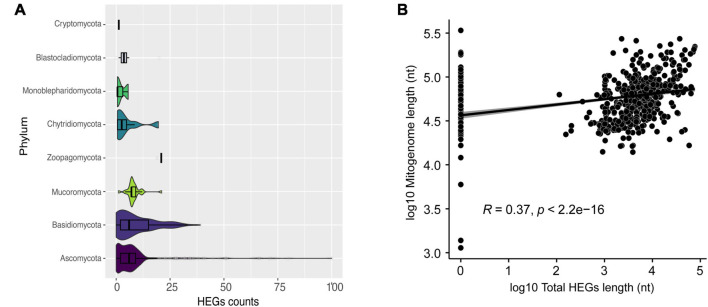
Characterization of HEGs. **(A)** Number of HEGs found in the mitogenomes by phylum. **(B)** Correlation between mitogenome length and total HEGs length.

In our analysis, we identified Ascomycota species that can have up to 100 HEG elements, such as *Nemania diffusa* (NC_049077). The other phyla exhibited a more homogeneous number of HEGs, with a mean of six HEGs per mitogenome (Figure 4A). HEGs have not been identified in many mitogenomes, such as *Acremonium fuci* (NC_029851) and *Hyaloraphidium curvatum* (NC_003048). Currently, six families of HEGs are known, with only two families found in fungal mitogenomes, LAGLIDADG and GIY-YIG (Hausner, 2003; Megarioti and Kouvelis, 2020). We identified a total of 4,432 LAGLIDADG and 1,247 GIY-YIG across all mitogenomes. Interestingly, we found 15 H-N-H elements, showing that these HEGs occur in the mitogenome of fungi, although they are rare (Hafez and Hausner, 2012; Megarioti and Kouvelis, 2020). H-N-H are usually found as free-standing or in group I introns (IGI) in Bacteria and their phages (Hafez and Hausner, 2012). All three aforementioned types of HEGs are able to cleave double-stranded DNA at specific sites, facilitating the mobility of the elements within the genome (Arbuthnot, 2015). Since numerous HEG elements were found in most mitogenomes of fungi, we asked whether there is a correlation between the mitogenome total length, and the total length of the fraction composed by HEGs. We found a significant positive Pearson correlation (*R* = 0.37; *p* < 2.2e-16) suggesting that the abundance of HEGs length is one of the factors responsible for the observed variation in fungal mitogenome size (Figure 4B). However, our data indicate that they may not be the only factor, leading us to investigate whether other non-coding elements would also be responsible for the variation in size and diversity in the composition of mitogenomes.

**FIGURE 5:**
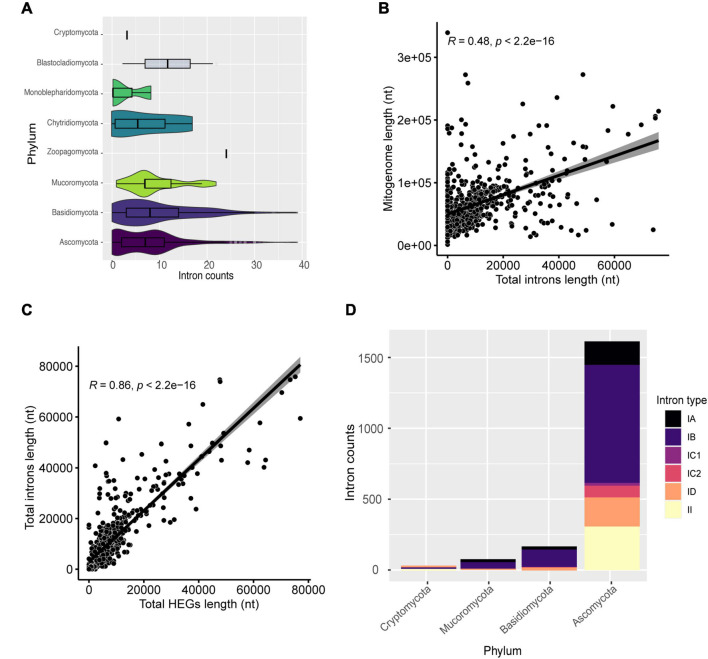
Characterization of introns. **(A)** Number of introns found in the mitogenomes by phylum. **(B)** Correlation between mitogenome length and total introns length. **(C)** Correlation between total introns length and total HEGs length. **(D)** The most frequent types of introns in the evaluated mitogenomes classified by phylum.

In total, 5,516 intronic sequences were identified. Ascomycota and Basidiomycota had 3,976 (72.1%) and 1,191 (21.59%) of all introns identified, respectively. Moreover, Ascomycota mitogenomes have an average of nine introns per mitogenome while the other phyla averaged less than five (Figure 5A). Mitogenomes with a high number of introns were identified, such as *Pyrrhoderma noxium* (Basidiomycota – CM008263) with 39 introns, *Phycomyces blakesleeanus* (Ascomycota- NC_027730) with 38 introns, *Parasitella parasítica* (Mucoromycota – NC_024944) with 22 introns, and *Rhizophydium* sp. (Chytridiomycota – NC_003053) with 17 introns. We found that the presence and global length of introns is correlated with the size of each mitogenome (*R* = 0.48, *p* < 2.2e-16) (Figure 5B). Many introns contain domains of HEGs, and since these elements can transpose to other parts of the genome, we investigated whether the length of introns was related to the HEGs length (Figure 5C), and found a correlation *R* = 0.86, which suggests that HEGs may be partly responsible for intron size, such as proposed previously (Edgell, 2009).

We assessed the percentage of HEGs within introns as well as the opposite (Supplementary Table 4). We found that 43.46% of all HEGs are within intronic regions (31.54% of GIY-YIG; 33.33% of HNH; and 46.99% of LAGLIDADG), and 48.23% of the analyzed introns overlap with HEGs coordinates, and, most frequently, LAGLIDADG (9.00% overlap GIY-YIG; 0.22% overlap H-N-H; and 39.01% overlap LAGLIDADG). In fact, these results agree with the literature, which shows that 30% of IGI contain internal ORFs that might represent HEGs (Chevalier and Stoddard, 2001; Hafez and Hausner, 2012). Most probably, introns originated before HEGs as first suggested by Loizos et al. (1994), providing a “safe haven” for these endonucleases to get established, although this theory does not explain why there is still many free-standing HEGs (Loizos et al., 1994). Edgell (2009) states that both HEGs and introns are selfish, independent elements, but they often benefit from being in close genomic proximity (e.g., HEGs are established by introns, and introns can use the endonuclease properties of HEGs to spread more easily) (Edgell, 2009). The association of HEGs with introns allows their persistence in the genome, since if they inserted themselves into genes, they would be subject to negative selective pressure to eliminate the selfish elements from the genome (Stoddard, 2014).

Generally, IGI is more common in fungi while IGII is more common in plants and most of them are self-splicing (Hausner et al., 2014; Fonseca et al., 2020). In this study, we performed the classification of identified introns and found six types (IA, IB, IC1, IC2, ID, and II). Introns from group IB were the most common among mitogenomes (562 or 76% of mitogenomes), while group IC1 was the least frequent (31 or 4.19% of mitogenomes). Moreover, when IGI is colonized by HEG domains, it can split a gene region due to the HEG cutting function (Lambowitz and Perlman, 1990; Belfort, 2003). Although IGII are usually infrequent, they were identified in 238 (32.21%) mitogenomes (Figure 5D). IGII introns have catalytic (ribozyme) and intron-encoded-protein sites, which allow their own splicing and proliferation in the genome (Lambowitz and Zimmerly, 2011). A previous study carried out by our group showed that intronic sequences can be shared among distinct species and at different genomic positions, indicating that the presence of self-splicing introns associated with maturases, such as HEGs, may be responsible for the transfer and sharing between genomes (Fonseca et al., 2020).

**FIGURE 6:**
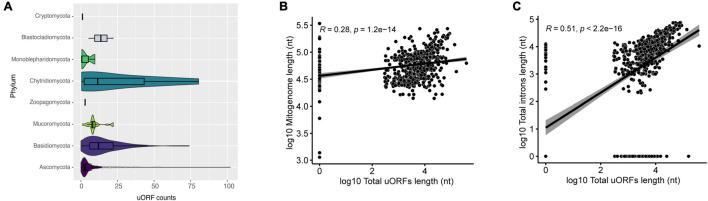
Characterization of uORFs. **(A)** Number of uORFs found in mitogenomes in each phylum. **(B)** Correlation between mitogenome length and total uORFs length. The correlation is in log10. **(C)** Correlation between total introns length and total uORFs length. The correlation is in log10.

When we evaluated the presence of uORFs in mitogenomes, species of Ascomycota had the highest number of uORFs. For example, *Morchella importuna* (NC_012621) had 102 uORF elements and a mitogenome length of 107 kbp (Figure 6A). Mitogenome and uORF lengths present a weak positive linear relationship (*R* = 0.28, *p* = 1.2 e-14) (Figure 6B), and a moderate positive linear correlation with introns length (Figure 6C). These uORF elements have been identified in different fungal mitogenomes, and some may be shared among species, as recently demonstrated (Fonseca et al., 2020; Araújo et al., 2021; Kulik et al., 2021), which suggests that uORFs within mitogenomes of fungi may play a significant role in shaping mitochondrial genome length. This is in agreement with a recent investigation of mitochondrial landscape in eukaryotes, which has found a positive correlation between mitochondrial genome length and the prevalence of unassigned regions (Formaggioni et al., 2021). Nonetheless, differences in the literature suggest that there are probably inter-phyla variations. For instance, the linear correlation coefficient is greater than 0.50 in both the Agaricomycetes class and the Hypocreales order (Fonseca et al., 2020; Araújo et al., 2021), a higher value than the one obtained in this study. As aforementioned, uORFs are not homogeneously present in all phyla analyzed, and therefore they may not influence mitogenome length at the same magnitude for different groups.

#### Diversity of Domains in uORFs

In our attempt to identify uORFs, we found that 64.49% of them have known protein domains (Supplementary Table 5) and most of them were sequences with HEGs domains (69.05%). We also identified some uORFs with protein domains related to plasmid genes: *dpo* (5.60%) and *rpo* (1.05%). A total of 35.51% of the uORFs lacked any similarity with known protein domains and may represent unidentified protein-coding genes. Nevertheless, by implementing a computational technique that assess coding potential based on sequence intrinsic features, 43.86% of uORFs were predicted to be coding (Supplementary Table 6). Since we were working with a kingdom-wide dataset, it was out of the scope of our study to conduct genus- or species-level transcriptomic analyses to verify if those uORFs are transcribed. When possible, this method can help shed light on whether uORFs are in fact transcribed or not, as demonstrated by Torriani et al. (2014) for some *Rhynchosporium* species (Torriani et al., 2014).

Mitochondrial plasmids are widely found in Fungi and some mitochondrial genes, such as *dpo* and *rpo*, are commonly associated with plasmid integration events (Formighieri et al., 2008; Férandon et al., 2013; Formaggioni et al., 2021). In our study, we identified *dpo* homologs in at least one species of six different phyla, with Basidiomycota and Ascomycota showing the highest numbers of sequences – 227 and 116, respectively (Supplementary Table 5). On the other hand, *rpo* genes were only annotated in Ascomycota (46 sequences), Basidiomycota (23), and Chytridiomycota (6). In total, amongst all mitogenomes analyzed, it was possible to observe that 10.74% of Ascomycota fungi presented a *dpo* and/or *rpo* homolog, in contrast with 57.14% of Basidiomycota species. For the other phyla, the lack of representatives makes difficult to draw meaningful conclusions. For instance, most Mucoromycota species in our dataset, have a *dpo* homolog; however, considering that we are working with only 24 Mucoromycota species, it is not a fair comparison with the most represented phyla Ascomycota and Basidiomycota.

**FIGURE 7:**
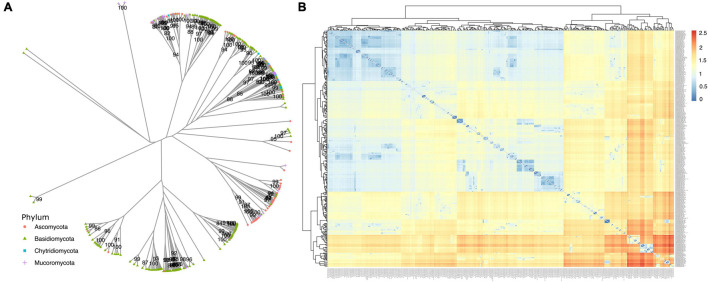
Phylogenetic analysis of *dpo* genes. **(A)** The phylogeny was constructed by the maximum likelihood method with 1,000 bootstrap replicates. Genes are classified by phylum. **(B)** A similarity matrix based on patristic distance. Dendrograms on top and left group related mitogenomes and distances are shown from closer mitogenomes (blue) to more distant ones (red).

**FIGURE 8:**
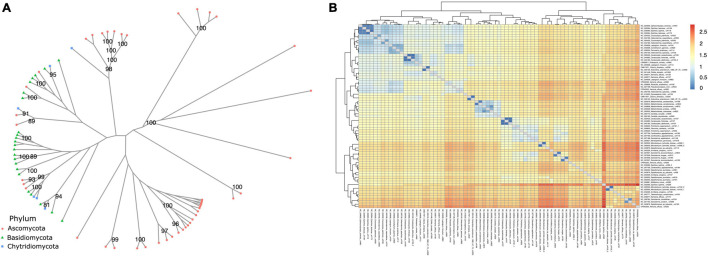
Phylogenetic analysis of *rpo* genes. **(A)** The phylogeny was constructed by the maximum likelihood method with 1,000 bootstrap replicates. Genes are classified by phylum. **(B)** A similarity matrix based on patristic distance. Dendrograms on top and left group related mitogenomes and distances are shown from closer mitogenomes (blue) to more distant ones (red).

When analyzing similarity among annotated *dpo* sequences (Figure 7), some form phyla-specific clades, whereas others seem to be shared among species of different phyla. A similar pattern was observed for *rpo* homologs (Figure 8). One possible explanation for this finding is horizontal gene transfer (HGT). As discussed by Beaudet et al. (2013), mitochondrial plasmids of fungi and plants exhibit many similarities, indicating that HGT events between organisms of those kingdoms may occur (Beaudet et al., 2013). According to Handa (2008) a plasmid from *Brassica* has an ORF with similarity to *rpo* from several organisms, including fungi (Handa, 2008). Moreover, since different species of fungi often share the same habitat, the hypothesis that HGT events between fungi occur is often discussed by many studies that identified highly similar *dpo* and/or *rpo* homologs among phylogenetically distant fungi (Wang et al., 2008; Xavier et al., 2012; Andrade and Goes-Neto, 2015).

#### Fungal Mitogenome Conformation and Genetic Code Usage

**FIGURE 9:**
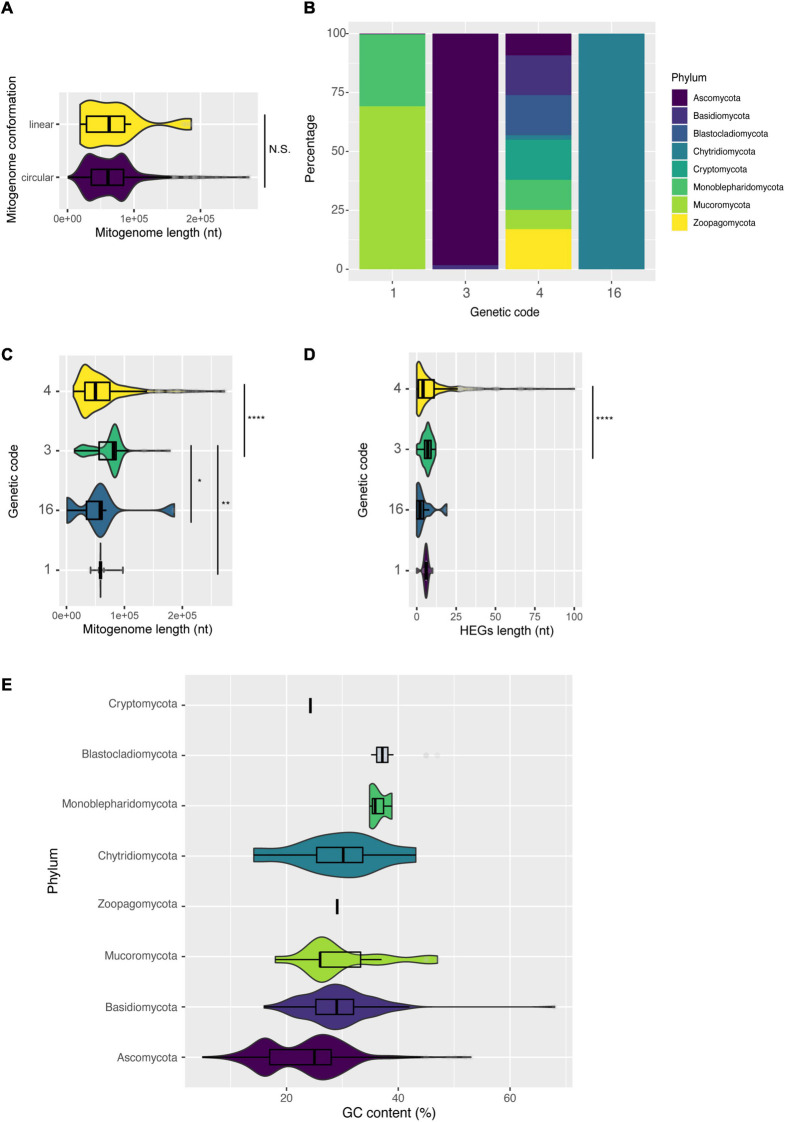
Characterization of mitogenomes according to intrinsic molecular characteristics of the mitogenome. **(A)** Length of mitogenomes by conformation: linear or circular. **(B)** Different genetic codes identified by phylum. **(C)** Length of mitogenomes by identified genetic code. **(D)** Number of HEGs according to the genetic code, and **(E)** variation of GC content among fungal phyla.

Although most mitogenomes are circular (Kulik et al., 2021), some genomes were annotated as linear. We wondered whether the conformation of the genome would affect its length, so we compared these values using Wilcoxon’s statistics and found no statistical difference (Figure 9A). Even though, the mitogenomes conformation had no effect in mitogenome length, fungal linear mitogenomes have distinct features when compared with circular mitogenomes, such as the presence of an invertron (terminal inverted repeats) and of one to six uORFs that may produce DNA or RNA polymerases (Handa, 2008). In our analysis, no invertron were detected in mitogenomes. The presence of linear mitogenomes has already been described in different eukaryotes, including fungi. One of the hypotheses is that linearization occurs due to the integration of plasmids containing genes that code for *dpo* (Williamson, 2002; van de Vossenberg et al., 2018). In our analysis, 37 (5.22%) linear genomes were identified and seven of them have *dpo* homologs. Examples are *Candida subhashii* and *Saccharomycopsi malanga*, with two *dpo* each. Despite the presence of these genes in some linear mitogenomes, the vast majority did not present *dpo*, suggesting that other mechanisms may be involved in mitogenome linearization.

Another little explored feature is the usage of different genetic codes in fungal mitogenomes. Among the 685 mitogenomes evaluated, the use of four different genetic codes was detected: code 1 (The Standard Code), code 3 (The Yeast Mitochondrial Code), code 4 (The Mold, Protozoan, and Coelenterate Mitochondrial Code and the *Mycoplasma*/*Spiroplasma* Code) and code 16 (Chlorophycean Mitochondrial Code)^6^. Overall, 62% of all mitogenomes use genetic code 4 (the UGA codon codes for Tryptophan), belonging to Ascomycota, Basidiomycota, Monoblepharidomycota, Cryptomycota, and Blastocladiomycota (Figure 9B). Most species of Mucoromycota are coded by code 1, while many species of Chytridiomycota are coded by code 16 (the TAG codon codes for Leucine) (Figure 9B and Supplementary Table 7). These results defy the classical ‘Frozen Accident Theory’ that proposes the immutability of the standard genetic code to avoid lethal genetic alterations (Crick, 1968). According to our genetic table analysis, most fungal species likely use the UGA codon (stop codon in standard genetic code 1) for translating Trp, similar to what was previously reported for UAA (Knight et al., 2001). Nonetheless, there is evidence that, in fact, a minority of fungal mitochondria uses UGA to code Tryptophan, showing that the annotation of fungal mitogenomes needs to be widely updated (Nibert, 2017). There are multiple theories to explain the differences of genetic code usage, but the one that better fits the fungal scenario may be related to the simplification of the mitochondrial genome and translation machinery (‘genome streamlining’ hypothesis), that would make the replication of mitogenomes easier, less energy costly and would also slowly simplify the mitogenome length (Andersson and Kurland, 1995; Knight et al., 2001; Swire et al., 2005).

We then compared the length of mitogenomes with different genetic codes (Figure 9C). Our results showed that fungi with genetic code 3 have larger mitogenomes than fungi with other genetic codes. According to NCBI Taxonomy^7^, the genetic code 3 classically represents yeasts. These unicellular fungi do not necessarily need mitogenomes for energy production, and they might have more accessory elements or non-coding regions, which contributes to the genomic size increase (Ruan et al., 2017). Many studies have demonstrated that the length of mitogenomes is generally affected by the presence of HEGs (Fonseca et al., 2020; Araújo et al., 2021), and we detected the same trend in our data when comparing genomes with genetic code 3 and 4 (Figure 9D).

We also observed a difference between the GC content of mitogenomes at phylum level (Figure 9E). Usually, fungal mitogenomes have a low GC content, and in our dataset the mean GC content was ∼25%. Ascomycota, Mucoromycota and Basidiomycota showed the highest variation. Some outliers can also be observed regarding GC content, such as *M. furfur* (Basidiomycota – CP046241) with 68% GC, some species of the genus *Candida* ranging from 13–53% (Ascomycota – NC_014337 and NC_022174), and the species *Glomus cerebriforme* with 47% GC (Mucoromycota – NC_022144). Some studies suggest that low GC content restricts mutations in mitochondrial genes (Grivell, 1989; Lang et al., 1999). Thus, mitogenomes that have a high GC content may be subject to higher mutational variations.

### Overrepresented Species and Global Network Analysis

Our findings indicated that the size of mitogenomes is associated with both the presence of accessory elements and protein-coding genes. Additionally, we observed that many mitogenomes from the same species (for instance, 29% of the analyzed mitogenomes are from 19 different species) remained after the similarity filter performed, and many do not have the 14 protein-coding and ribosomal core genes (74% of the mitogenomes from the same species lack at least one core gene). These aspects limited the use of a phylogenetic analysis exploring the evolutionary relationships among species, since there were mitogenomes without any gene in common to be aligned. Thus, we performed similarity network analyses to investigate the relationships between the evaluated mitogenomes.

The first analysis was done using only species that had more than three different mitogenomes. Divergent mitogenomes were found in more than one phylum. Three main groups of mitogenomes can be cited, those from the species of the genus *Saccharomyces*, *Fusarium*, and *Aspergillus*. The genus *Saccharomyces* is one group of fungi that exhibited more than one divergent mitogenome, which after the 90% similarity filter, retained 149 mitogenomes (out of 166 mitogenomes – 10.24% reduction). *Saccharomyces* can produce energy via the functioning of the mitochondrial organelle or fermentation. Therefore, these organisms can survive only with fermentable carbon sources and even without the presence of a mitogenome. These yeasts present a different phenotype, described as “petit format,” unable to grow on non-fermentable carbon sources (Bulder, 1964; Goldring et al., 1970; Day, 2013) and their mitogenomes may suffer a slightly different selective pressure in relation to other groups of fungi that depend on mitochondria to grow. Furthermore, the length of mitogenomes of *Saccharomyces* can vary between two- and four-fold (Borneman and Pretorius, 2015), and this variation may be related to the expansion of intergenic regions already described in this group of organisms (Ruan et al., 2017).

**FIGURE 10:**
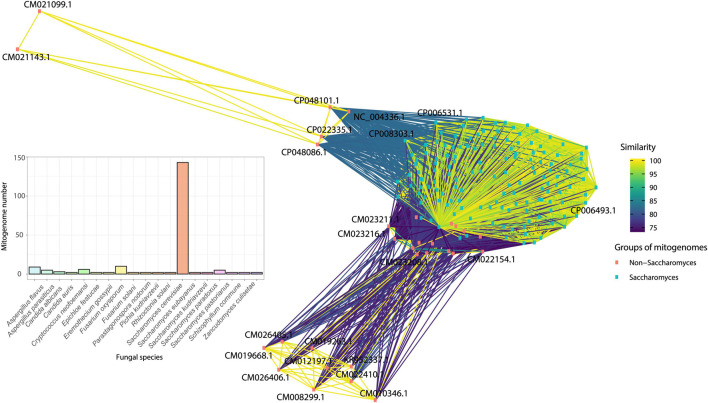
Similarity network of species that displayed more than two mitogenomes after filtering the database using CD-HIT. Mitogenomes of *Saccharomyces* species have a high similarity and are pink squares, while the other duplicated mitogenomes are blue squares. The bar graph shows the total number of different mitogenomes of each species in the network.

Another species that also had divergent mitogenomes is *Aspergillus flavus*, for which nine mitogenomes remained after redundancy-filtering, with mitogenomes varied between 29 and 31 kbp. Similarly, the dimorphic *Cryptococcus neoformans* continued with six mitogenomes and size between 24 and 30 kbp. The species *Fusarium oxysporum* presented ten mitogenomes and sizes ranging from 34 to 52 kbp. Through the sequence similarity network, we can observe the formation of three main clusters (Figure 10). The first cluster, composed of mitogenomes of the genus *Saccharomyces* (green dots on bottom), the second one containing mitogenomes only from the species *F. oxysporum* (orange dots on top right) and the third, containing only the species *C. neoformans* (orange dots on top left). In this last group, it was possible to observe that some mitogenomes of *C. neoformans* (CP048101, CP048086, CP022335, and NC_004346) were grouped with a closer proximity to mitogenomes of the genus *Aspergillus* (*A. flavus* and *Aspergillus parasiticus*). In general, no significant difference in mitogenome length was observed and all *Cryptococcus* mitogenomes present all core genes. A mitogenome of *F. oxysporum* (NC_017930) also grouped with the *Aspergillus* genus cluster. This mitogenome is the smallest of all *F. oxysporum* available (34 kbp). Size variation and differences in gene composition in the *F. oxysporum* species has been described and are associated with the presence of intronic sequences in the *nad5*, *cob* and *atp6*. Indeed, Brankovics et al. (2017), classified *F. oxysporum* strains into clades according to the presence of these sequences (Brankovics et al., 2017). The same observation was made for species of the genera *Aspergillus* and *Cryptococcus*, in which the presence of accessory elements such as introns and uORFs are responsible for the increase in size of the mitogenome (Joardar et al., 2012; Wang and Xu, 2020).

**FIGURE 11:**
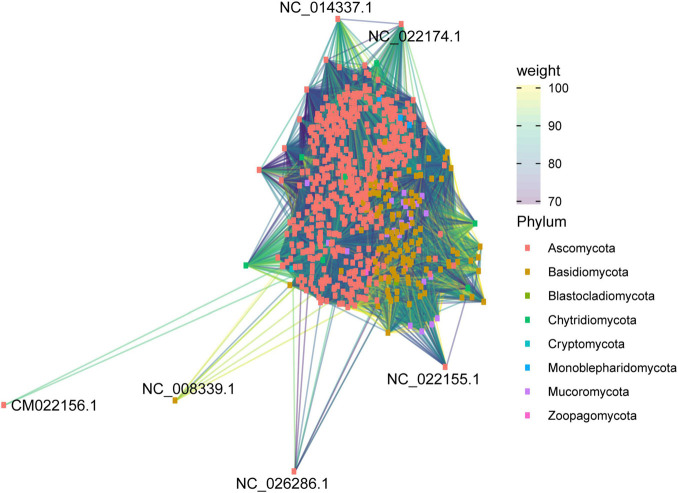
Similarity network of all mitogenomes evaluated in this study. Mitogenomes are classified by fungal phylum.

The second network analysis considered all mitogenomes evaluated to assess the grouping according to phylum-level classification. The clustering patterns among the evaluated mitogenomes are displayed in Figure 11. Many species were grouped according to their respective phylum, indicating that the variation in the composition of mitogenomes is lower than the similarity between the taxonomic groups of fungi. The abundance of Ascomycota and Basidiomycota mitogenomes in relation to the other phyla can also be observed. In some cases, we found clustering among different phyla, such as Ascomycota and Mucoromycota. This grouping may be due to the presence of accessory elements that can be shared between species from different phyla. In this study, phylogenetic analysis indicated that *dpo* homologs were shared between Ascomycota and Mucoromycota species (Figure 8), confirming that these elements may also be responsible for the grouping of mitogenomes. Other exceptions are also presented. The species *Alternaria alternata* (Ascomycota – CM022156) was distant from the other mitogenomes of the same phylum. According to our annotation, this species has 23 uORFs and only the ribosomal genes *rns* and *rnl*, which may be one of the reasons for the distance among this mitogenome and the others from Kingdom Fungi. Furthermore, Chytridiomycota representatives were not well grouped. The Chytridiomycota are pathogenic and saprotrophic fungi commonly found in freshwater environments. This phylum presents some systematic controversies about the permanence of some species in the group (Levin, 2013), and this fact may help explain the lack of grouping among the evaluated mitogenomes.

## Conclusion and Perspectives: Why to Study Fungal Mitogenomes?

Studies that fully characterize the structure of fungal mitogenomes are still scarce. In the current study, we explored the diversity of genome structure of fungal mitogenomes at kingdom level and demonstrated that less than 0.02% of the already described fungal species have their mitogenome sequenced and publicly available. The phylum Ascomycota has the highest number of mitogenomes available, while some other phyla do not have even one single mitogenome sequenced. Taken together, our findings suggest that fungal mitogenomes exhibit high structural variation (composition, genetic codes, size, and gene diversity). The size and composition of mitogenomes were variable, mainly explained by the presence of accessory elements. Furthermore, there was no universal gene found in all fungal species. Some studies have already demonstrated the possibility of using mitochondrial genes to identify fungal species (Kulik et al., 2015; Smith, 2015; Avin et al., 2017). Nevertheless, our work indicates that there is no universal marker, which does not prevent the use of mitochondrial genes to identify groups of fungi at the order or family levels.

Our findings clearly show that, despite being considered conserved, fungal mitogenomes have numerous differences that need to be better characterized and understood. Because of this structural diversity, the use of mitogenomes are a promising tool for elucidating evolutionary relationships between species as they have a low rate of recombination, diverse patterns of mitochondrial inheritance, difference in mutation rate in relation to the nuclear genome, and facility to be amplified by PCR to sequence a particular region or the entire mitogenome due to the high number of copies in the cellular cytoplasm (Rubinoff and Holland, 2005).

Despite the important role played by mitochondria in the cell, detailed knowledge about the gene regulation of this organelle is still scarce, and very little is known about the control of expansion/contraction of mitochondrial genomes in fungi (Dirick et al., 2014; Nguyen et al., 2020; Kulik et al., 2021). The sequencing and characterization of new fungal mitochondrial genomes coupled with transcriptomic studies are of great importance to help understand genome function and gene regulation of this organelle (Grahl et al., 2012; Mosbach et al., 2017; Tang et al., 2018). Therefore, the deep characterization of fungal mitogenomes at kingdom level presented here provides a first step toward these goals.

In a more applied approach, the mitochondrial genome may also provide new strategies for controlling fungal pathogens, as well as may help improve the production of secondary metabolites (Grahl et al., 2012; Mosbach et al., 2017; Tang et al., 2018), since fungal mitochondria participate in several processes of pathogenicity, virulence, and drug resistance. Fungicides usually target proteins located in the mitochondrial inner membrane or mitogenome (Song et al., 2020). Furthermore, these studies still help investigate the function of mitochondria in cellular processes that are essential for the growth and development across fungi. To perform the characterization of resistance mutations, it is necessary to sequence and make available numerous mitogenomes from the same species.

We detected a large amount of mitogenomes available for Saccharomycetaceae, showing a representative and non-redundant dataset. This genetic variation in mitogenomes and phenotypic diversity observed especially in *Saccharomyces* makes the genus a good model for studies of comparative mitogenomics. Currently, most of the knowledge on comparative genomics in fungi comes from studies within Saccharomycetaceae (Freel et al., 2015; Peter et al., 2018), but mitogenomes are still not explored in populational, ecological, and evolutionary studies.

We also identified many unusual genes in mitogenomes, such as *dpo*, *rpo*, and mitochondrial genes usually located in the nuclear genome. This gene diversity may be related to HGT between species and even mitogenome length (Rosewich and Kistler, 2000; Joardar et al., 2012; Kolesnikova et al., 2019; Fonseca et al., 2020; Araújo et al., 2021). It is even more notable when we evaluate different mitogenomes of the same species with different uORFs and genes, as observed for *Fusarium* and *Saccharomyces* species, indicating that fungal mitogenomes have a great plasticity. The implications of the presence of these genes are still poorly understood. Until December 2020, few fungal species had more than one sequenced mitogenome, preventing intraspecific comparisons. This scarcity of genomic data hinders the search for new antifungal agents and the characterization of resistance mutations, as well as the understanding of fungal infections in different hosts. As a summary, the results found in this study show that fungal mitogenomes are highly diverse, without the presence of any universally conserved gene, and vary widely in accessory elements, suggesting that they are in constant and fast change.

## Data Availability Statement

The original contributions presented in the study are included in the article/Supplementary Material, further inquiries can be directed to the corresponding authors. All scripts used in our study are available at: github.com/LBMCF/fungal_mitogenomes_ frontiers.

## Author Contributions

PF, RD-P, DA, LT, TM-P, WR, L-ED-B, EA, and AG-N analyzed the data and wrote the manuscript. All authors read and approved the final manuscript.

## Conflict of Interest

The authors declare that the research was conducted in the absence of any commercial or financial relationships that could be construed as a potential conflict of interest.

## Publisher’s Note

All claims expressed in this article are solely those of the authors and do not necessarily represent those of their affiliated organizations, or those of the publisher, the editors and the reviewers. Any product that may be evaluated in this article, or claim that may be made by its manufacturer, is not guaranteed or endorsed by the publisher.

## Footnotes

1
https://www.ncbi.nlm.nih.gov/genome/browse#!/organelles/

2
https://www.ncbi.nlm.nih.gov/Taxonomy/Browser/wwwtax.cgi

3
http://megasun.bch.umontreal.ca/cgi-bin/mfannot/mfannotInterface.pl

4
https://www.ncbi.nlm.nih.gov/

5
https://www.ncbi.nlm.nih.gov/cdd/

6
https://www.ncbi.nlm.nih.gov/Taxonomy/Utils/wprintgc.cgi

7
https://www.ncbi.nlm.nih.gov/taxonomy

## Supplementary Material

Supplementary Table 1 Overview of studies assessing fungal mitogenomes published from 1990 to 2020. Title and year of publication are shown for the 481 studies.

Supplementary Table 2 Number of mitogenomes filtered with CD-HIT at thresholds of 99–90, 80 and 70% of identity. The reduction value in regarding the total number of mitogenomes is also shown.

Supplementary Table 3 Detailed description of fungal mitogenomes used in this study. Accession numbers, taxonomy, mitogenome size, number of accessory elements and genome conformation are available.

Supplementary Table 4 Overview of introns and HEGs identified in the mitogenomes assessed in this study. Introns that have domains of HEGs are also shown.

Supplementary Table 5 Presence of conserved domains in uORFs sequences. The analysis was performed on the CDD database with standard parameters.

Supplementary Table 6 Coding potential of uORFs. The coding potential was calculated with the CPC2 program with standard parameters.

Supplementary Table 7 Differences between the use of codons and amino acids according to the four genetic codes identified in fungal mitogenomes.

Supplementary File 1 *de novo* annotation files of the 685 mitogenomes investigated in this study. Annotation was performed with the Mfannot program.

Supplementary File 2 Domains annotated in the uORF sequences of the *Malassezia furfur* mitogenome. The analysis was performed using the CDD database.
